# Annular Cavity Design for Photoluminescent Polymer Optical Fiber Sensors

**DOI:** 10.3390/s20185199

**Published:** 2020-09-11

**Authors:** Rune Inglev, Jakob Janting, Ole Bang

**Affiliations:** Department of Photonics, Danish Technical University, Ørsteds Plads Building 343, 2800 Kongens Lyngby, Denmark; runeinglev@gmail.com (R.I.); oban@fotonik.dtu.dk (O.B.)

**Keywords:** polymer optical fibers, photoluminescence, sensor design

## Abstract

We present optimization results on the design of a polymer optical fiber single point sensor suitable for photoluminescence-based sensing. The single point sensing design consists of one or two annular cavities, separated by a small distance, milled into the fiber and subsequently filled with a thick solution of polymer, solvent, and photoluminescent molecules, which is then allowed to dry. The design is tested by varying the depth and length of a single cavity and utilizing two cavities with varying separations. Results from experiments show a maximum response at a separation of 2 mm for which we present an analytical explanation. A geometrical, numerical simulation model, taking into account both skew and meridional rays, is developed and shows very good agreement with the experimental results. The fiber design presents a general platform that has the potential for the fabrication of multi-point photoluminescent sensors, for which it is necessary to have several points along the fiber functionalized for sensing. Furthermore, the approach with polymer fibers and polymer sensing gels allows for a robust integration of the sensing matrix and the optical fiber, more so than is possible using glass optical fibers.

## 1. Introduction

The photoluminescence-based optical detection of various compounds has been around for approximately a century. The fluorescence microscope is probably the first application of photoluminescence in an analytical sense since its invention in the early 1910s by Heimstadt and Reichert [1]. The theories underlying photoluminescence were not completely understood in the early 20th Century, but still found useful applications in other areas than microscopy, such as the detection of uranium [2], spores [3], oxygen [4,5], and more. In the early 1940s, Coons et al. developed the technique of using fluorescent antibodies [6], and some of the first applications were in the detection of diseases and bacteria [7,8].

Optical sensors using photoluminescence can have many different designs. Some developments have been in the direction of special indicator spots, for example for oxygen detection in, e.g., bioreactors [9]. Other designs are based on the use of optical fibers in conjunction with photoluminescent compounds [10,11,12].

One of the reasons to use optical fibers as the sensing platform is the ability for remote sensing, which was pointed out early in the history of fiber optical sensing [13]. The main contribution of the optical fibers is to act as waveguides, transporting the excitation and emission light to/from the sensing point. The sensing point has often been placed at the distal end of the fiber when it comes to photoluminescent sensors, although there are many examples of designs for in-line sensors with the capability of sensing at particular points along the fiber axis [14,15,16,17].

When using optical fibers, the choice stands between using a fiber made from either polymer or one of glass. Glass optical fibers have the definitive advantage when it comes to transmission over longer distances, but polymers allow for high flexibility due to a lower Young’s modulus (thirty times smaller than silica) [18]. Furthermore, in many sensing applications, long distances are not required, and polymer fibers find their justification. Polymer Optical Fibers (POFs) have been used in many different sensing applications to date. With Fiber Bragg Gratings (FBGs), POFs have been used for strain/force [19,20,21,22], humidity [23,24,25], and temperature [26] sensing. Using tapering, POFs have been modified for fiber-optical oxygen sensors based on photoluminescence [12], and by suitable chemical functionalization with antibodies of the polymer, a microstructured POF has been used for fluorescence-based localized biosensing [27,28,29].

The modifiability of the polymer fibers is one of their core advantages in the development of sensors. With suitable UV or Vis light, it is possible to inscribe FBGs into the fiber core, even if the core is thick [30,31]; using simple solvents, one can etch polymer fibers locally for various applications [16,32,33,34,35,36], and by suitable selection, it is possible to strongly integrate polymers with each other or apply polymer gels [37,38], a technique used in this paper.

**Figure 1:**
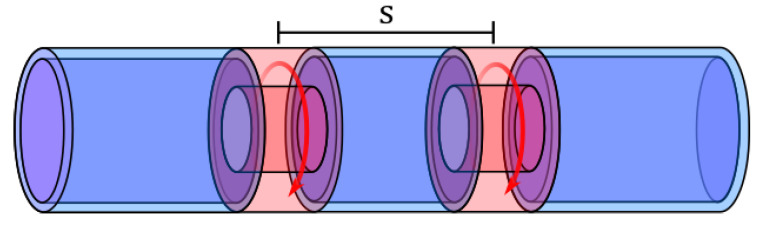
The concept of the Polymer Optical Fiber (POF) sensor. Annular cavities are milled in the fiber at specific locations. These cavities are then filled with a gel solution of PMMA and solvent, containing photoluminescent molecules quenchable by oxygen. The solvent evaporates and leaves a PMMA matrix with embedded photoluminescent molecules.

In this paper, we present a new photoluminescent sensor concept. A POF is modified by mechanically milling two annular cavities, separated by a small distance, at a specific position along its length. The cavities are then filled with a specialized solution of polymer, photoluminescent compound, and solvent (see Figure 1). As the solvent evaporates, the solution solidifies into a volume of polymer, solvent bonded (molecular entanglement) to the fiber and containing photoluminescent compound. Oxygen can diffuse into the volume and affect the photoluminescence by quenching. The signal from the sensing point will be the sum of the signal from each of the two cavities.

By creating an annular cavity, light is still allowed to pass through the sensing point and further down the optical fiber; yet, it will have a stronger interaction with the electromagnetic field than an evanescent wave sensor embedded in the cladding. There will still be loss associated with absorption and scattering, but it is likely to be less than experienced for designs in which a hole is drilled through the fiber [39,40]. Some designs have, instead of drilling all the way through the fiber, focused on creating several shallow cavities after each other, which could then be filled with a sensing gel [41]. That design also works by utilizing the aggregation of signals from several closely-spaced cavities.

By allowing the light to pass, the sensor concept has potential for multi-point sensors, such as described by Eich et al. [17], in which it is necessary to have several functionalized points along the fiber capable of local sensing, and thereby create a profile of one or more measurands along the length of the fiber. One could imagine having one sensing point located at one position on the fiber, consisting of two closely-spaced annular cavities, and another sensing point located further down the optical fiber, with another two closely-spaced annular cavities. By using annular cavities, the light can pass both to and from the second sensing point and so on further down the fiber.

Our initial assumption was that creating a single sensing point by having two cavities would result in a stronger photoluminescence signal than a single cavity (of the same size as either of the two). However, our goal has also been to avoid creating a long cavity (along the length of the fiber), which would also provide a stronger signal, since long cavities would be difficult to fill evenly with a gel. Besides, a long, deep cavity will make the fiber more fragile. Using smaller cavities placed after each other was assumed to have the same effect as a single long cavity in the aggregate, while also maintaining the mechanical integrity of the fiber.

An important parameter for any optical sensor using photoluminescence is the intensity of photoluminescence from the sensing point. Precise measurement of oxygen levels requires intensities that allow the electronics in the reader-unit to function optimally. Higher intensities are easier to develop reader solutions for and can also mean cheaper development costs. At the same time, the design must be such that a suitable fabrication process can be found.

This paper presents results on the evaluation of the concept described in Figure 1, by varying the depth and length of a single cavity and utilizing more than one cavity with varying separations. We first present the experimental results and move on to provide an analytical answer to some of the observed data. Finally, we will show the results from a numerical model simulation, in an attempt to recreate the experimental findings.

## 2. Materials and Methods

### 2.1. Materials

#### 2.1.1. Fibers

The optical fibers used are 1 mm thick PMMA/PVDF (core/cladding) fibers acquired from Edmund Optics (Product No. 02-534). The fibers are manufactured by Mitsubishi (ESKA CK-40). The core of the fibers has a diameter of approximately 980 μm. The cladding is approximately 10 μm.

#### 2.1.2. Measurement

The measurements were performed using an Ocean Optics HR2000 spectrometer. Simple color filters from LEE Filters were used for the removal of excitation light.

#### 2.1.3. Chemicals

The solvents and photoluminescent compound, PtOEP (Platinum Octaethylporphyrin), were acquired from Sigma Aldrich. PMMA (from GEHR GmbH) was dissolved in a mixture of 25% acetone and 75% trichloroethylene. The gel mixture was a 15% (v/v) solution of PMMA. PtOEP was added to the mixture, enough to create a molar concentration of 0.77 mM. The chemicals both had grades of >95%.

### 2.2. Methods

#### 2.2.1. Cavity fabrication

**Figure 2:**
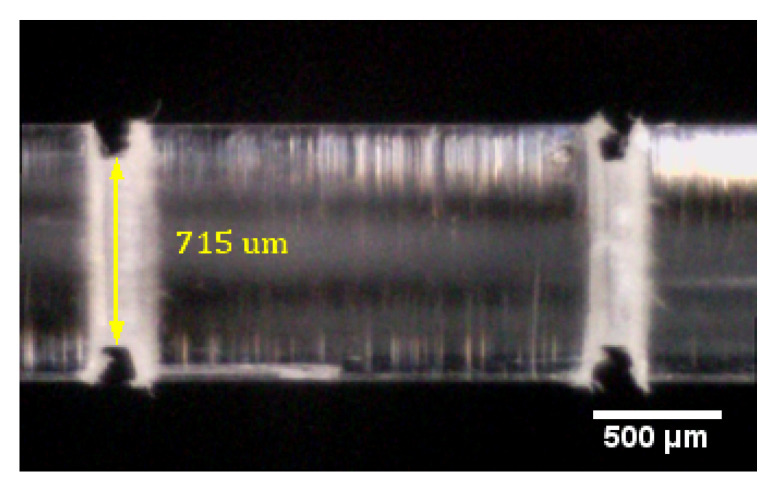
Cavities milled in the fiber. The fiber has a diameter of 1 mm, and the cavity depth is 137.5 μm. The separation of the cavities is approximately 2 mm.

**Figure 3:**
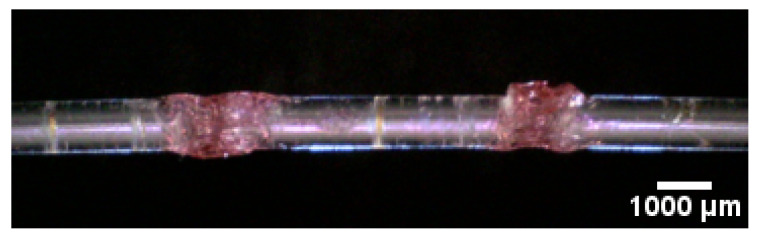
Example of a fiber with two annular cavities. The initial trenches were filled with the gel solution of solvent, polymer, and Platinum Octaethylporphyrin (PtOEP) and left for 1 day to solidify. The fiber is 1 mm in diameter, and the separation of the cavities is 5 mm.

The fibers were first milled using a Proxxon MICRO Mill MF 70. The milling was performed using a triangular milling head with a speed of 20,000 RPM. The fibers were fixated in a setup that allowed them to be rotated around the fiber axis while the miller was running. After milling, the fibers were annealed for at least 16 h at 90 C and 90% relative humidity. This served to remove any residual stress in the fiber, especially at the sensing point [24]. An example can be seen in Figure 2. Residual stress can cause the fiber to break when the gel solution is added. The gel was applied to the sensing point, and the fibers were left to dry for a day, during which most of the solvent left the mixture [35]. All fibers of a given batch were fabricated on the same day, to compare them later. An example of a filled and dried sample is given in Figure 3.

#### 2.2.2. Photoluminescence response

**Figure 4:**
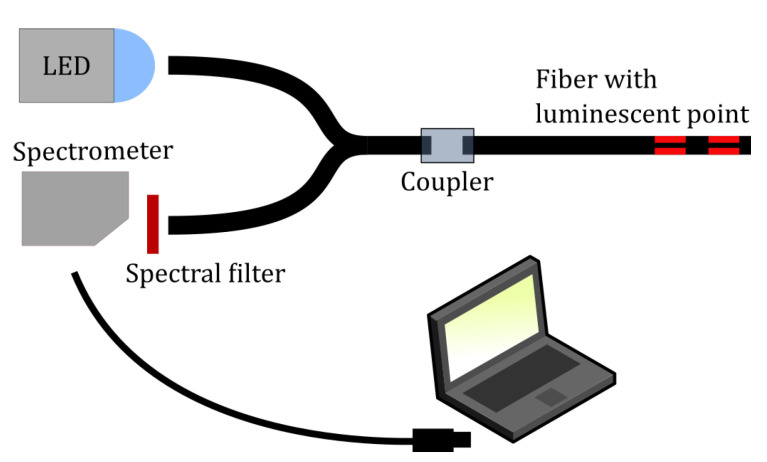
Experimental setup. Light from an LED (470 nm) is coupled into a 1 mm POF splitter (acquired from FiberFin). The light is guided into a sample fiber with the photoluminescent annular cavities via an SMA coupler. The light returning will be guided back into the splitter, and some of it will travel to the spectrometer. Before entering the spectrometer, the light passes through a spectral filter, removing the LED excitation wavelength. The spectra are acquired via a laptop.

For a given batch of fibers to be compared, all of them must have been manufactured on the same day and given an equal amount of drying time. We found that the photoluminescent response increases in the first few days after applying the sensing solution, possibly as a result of the evaporation of residual solvent molecules, thus leaving a more rigid medium for the phosphorescent PtOEP and thereby a higher quantum yield [42]. It is therefore important that fibers to be compared be measured after an equal number of days from fabrication. By “batch”, we mean the collection of fibers with differing geometrical parameters (1 cavity, 2 cavities, depth, width, length, separation), which will be compared relative to each other. The measurements were performed in the setup as portrayed in Figure 4. The functionalized optical fiber with one or more photoluminescent annular cavities was connected via an SMA coupler to a fiber-splitter. Excitation light form a blue LED was coupled into one arm of the splitter. The second arm of the splitter went to an optical filter capable of removing any reflected excitation light and only letting the photoluminescent emission to pass. The photoluminescence signal then entered the spectrometer. The integration time of the spectrometer was set to 2000 ms.

## 3. Experimental Results

Three different experiments were conducted. In the first two, only a single cavity was created, and the depth, *d*, and length (along the fiber), *l*, of this cavity were varied to see how the response behaved. In the first experiment, the cavity depth was varied while keeping the length constant. In the second experiment, we varied the length of the cavity and kept the depth constant.

The third and final experiment was with two cavities. However, in this experiment, a number of fibers with a single cavity were also created and measured, in order to get the relative gain of adding a second cavity. This would also allow us to compare the three experiments in terms of how each of them compared to a single cavity.

**Figure 5:**
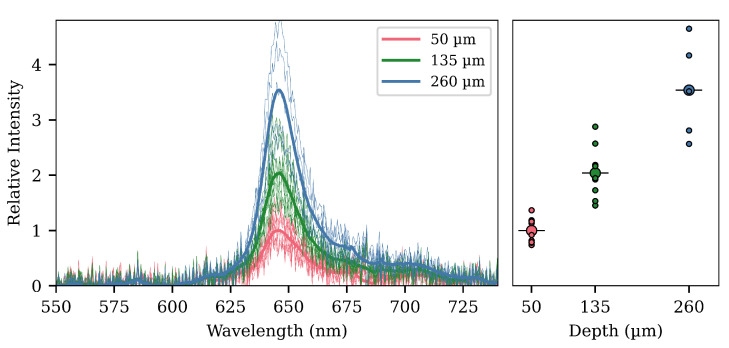
Photoluminescence spectra and signal response from the cavity-depth experiments. The intensities are shown relative to the average of the intensity for the 50 μm batch. The results clearly show an increase in the photoluminescence signal with increasing depth (the cavity length is fixed at approximately 140 μm). To the left, the recorded spectra are shown with the thick lines representing the average of a given batch. In the graph on the right side, the relative peak intensity is shown for all the different fibers. The larger circle with the horizontal line through is the average for the batch, while the smaller circles are the individual fiber responses. The peak just above 680 nm is a result of defective pixels on the CCD spectrometer.

**Figure 6:**
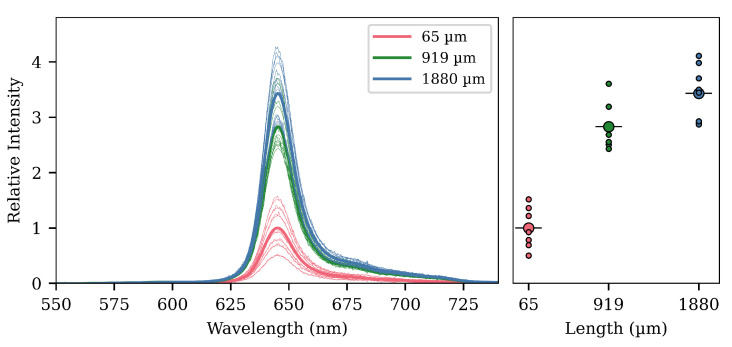
Results of varying the cavity lengths and with fixed depths at about 140 μm. The signal increases rapidly from 65 μm to 1 mm, but slows between 1 and 2 mm. The plot to the right shows the peak intensities of the spectra. The larger circle with the horizontal line through is the average for the batch, while the smaller circles are the individual fiber responses. The peak around 680 nm is a result of defective pixels.

In Figure 5 and Figure 6, presenting the results, a black, dotted line is also plotted for the simulation model developed. We will comment more on this model in Section 5.

### 3.1. Effect of Cavity Depth

Three different depths of d= 50, 135 and 260 μm were tested in order to determine the influence of this parameter on the photoluminescent response. The lengths of the cavities were approximately 140 μm. Ten fibers of each type were fabricated, and of the ten fibers with a depth of 50 μm, all remained, while nine remained of the ones with 135 μm cavities. Only five fibers with the deep 260 μm cavities remained. The fibers that did not survive broke at the cavity after the gel mixture was applied, probably because of Environmental Stress Cracking (ESC) [43], even though the fibers had been pre-annealed. The cause for this is most likely that the solvent is aggressive for the polymer core of the fiber. Any stresses, either internal or external, can easily break the thinned and plasticized core during the drying process. From these results, it could be concluded that cavity depths should stay well below 260 μm, and possibly even below 200 μm, to avoid breaking the fiber when the gel is applied.

The surviving fibers were investigated in the experimental setup previously mentioned. The results of these measurements can be seen in Figure 5, in which the clear trend is that a deeper trench will give rise to a stronger response. This is intuitive, as the excitation light will have a larger surface area with which to interact. However, as we have noted, while a deeper cavity will give a stronger signal, the fabrication will be more difficult due to the thin core and possible ESC.

### 3.2. Effect of Cavity Length

The second experiment looked at the increasing photoluminescent signal through longer cavities. Three lengths were tested, averaging l= 0.065, 1 and 1.9 mm, and all with d= 140 μm. Eight fibers for each length were fabricated. The results are depicted in Figure 6. There is an increase in signal strength with increasing length. However, the gain diminishes with length. As with the depth of the cavities, it is more difficult to fabricate longer cavities than shorter ones. Furthermore, filling short cavities is easier, as the gel required can be dispensed in a single blob very easily. Thus, from a fabrication point of view, it is therefore of interest to keep the cavities short, while still meeting the necessary requirements for the signal strength.

The diminished gain at longer lengths could be due to a self-screening effect of the cavity. The light originating from the far end of the cavity first must traverse the cavity itself, before propagating in the optical fiber back toward the detector. This light is partially blocked by the cavity itself if the cavity is long.

### 3.3. Effect of Dual Cavities

The third experiment investigated the effect of adding a second cavity. It would be intuitive to assume that an extra cavity will result in an increase in signal. If the two cavities were close enough together that they were effectively one cavity with double the length, then we know from the earlier experiment that this is already better than a single cavity. By separating the cavities, the second cavity presents a new surface, perpendicular to the fiber axis, available as a photoluminescence source.

**Figure 7:**
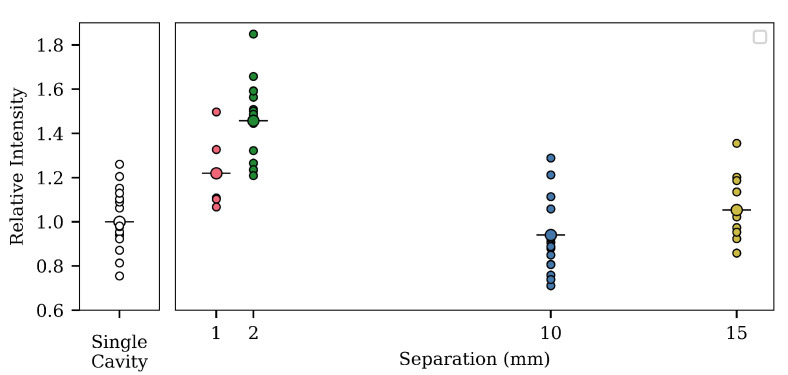
Photoluminescence intensity from dual cavities. The intensities are shown relative to the average intensity for a reference batch of single-cavity fibers (left-most plot) with 0.15 mm cavity lengths. In the graph on the right side, the relative peak intensity is shown for all the different fibers. The larger circle with the horizontal line through is the average for the batch, while the smaller circles are the individual fiber responses. The cavities had average depths of 125 μm and average lengths of 130 μm. The left most plot shows the variation of the single-cavity fibers. Ideally, the reference batch and dual-cavity fibers would have had the same cavity dimensions, but the fabrication method made this difficult. However, the goal is to show the relative impact on dual-cavity separation. The trend shows a maximum existing at 2 mm, where after, the addition of a second cavity has less impact.

In Figure 7, we see the results from the experiment, in which cavities with depth 125 μm and length 130 μm are made with varying separations. Even though the variance is large within the samples of a given separation, a trend is clear. With a 1 mm separation, the average intensity is more than 1.2 times the single-cavity intensity, and at 2 mm, the average intensity is around 1.5 times the single-cavity intensity. However, further out, at 10 and 15 mm, the average intensity once again drops and becomes comparable, or smaller, than the intensity from the 1 mm separation. These results point toward the existence of a maximum somewhere between 1 and 10 mm.

In the following sections, we will analyze this idea of an optimal separation, first by an analytical approach considering the meridional rays in the fiber and, next, using a numerical model taking into account both skew and meridional rays, as well as other effects.

## 4. Results from Meridional Analysis

To explain some of the results from the the experiments, we will analyze the design by a geometrical optics approach. The reason for this is that that all the length scales in the design are several orders of magnitude larger than the wavelength, and the fiber is a heavily multi-mode fiber.

**Figure 8:**
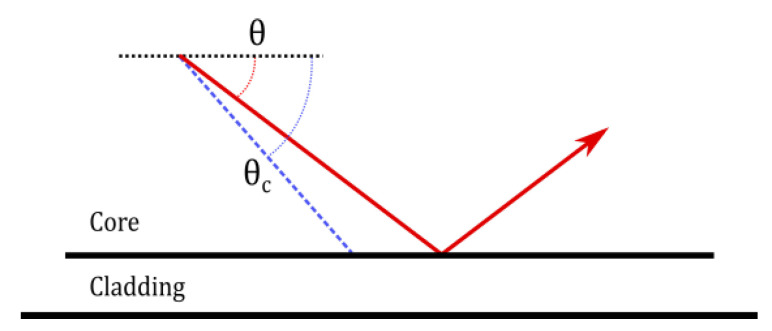
Polar angle and maximum polar angle $\theta_{c}$ as measured from the fiber propagation axis.

In the analysis, we assume that optical rays exist for all angles less than or equal to the complementary angle to the critical angle for Total Internal Reflection (TIR); this angle we will call the polar angle, θ (see Figure 8). The numerical aperture, NA, of the fiber is given by the manufacturer as 0.51, and the core material is PMMA with a refractive index of 1.49. We calculate the maximum polar angle, $\theta_{c}$, with Equation (1). Rays with polar angles larger than $\theta_{c}$ will not undergo TIR at the core-cladding interface.

(1) $$\theta_{c}=\frac{\pi }{2}-\sin^{-1}\left(\sqrt{1-\frac{{\mathrm{NA}}^{2}}{n_{core}^{2}}}\right)\approx 20^{\circ }$$

**Figure 9:**
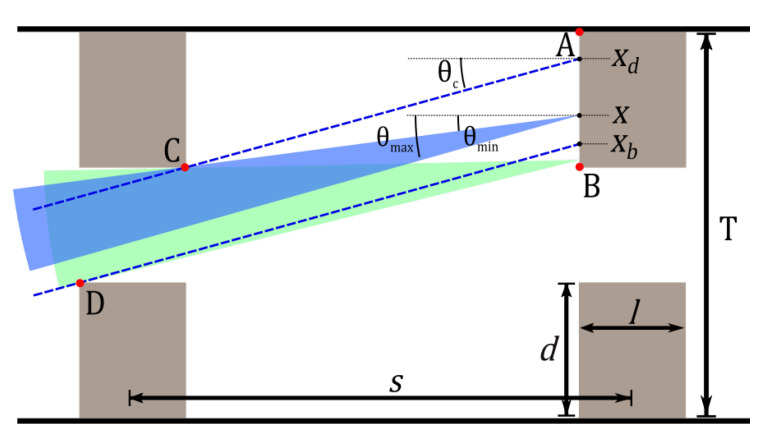
The meridional picture of the two-cavity design. A point *x* on the surface A-B is reachable through an aperture, presented by the left-most cavity. Light rays with polar angles in the sector bounded by $\theta_{min}$ and $\theta_{max}$ are able to reach and excite the point, as well as be emitted from the point and make it to the detector. The angles $\theta_{min}$ and $\theta_{max}$ must be smaller than or equal to the maximum polar angle of $\theta_{c}$. A ray originating from between Point B and the point $x_{b}$ will have its maximum angle constrained by Point D.

The situation we will analyze is depicted in Figure 9, in which the two annular cavities are seen in a meridional plane of the optical fiber. The cavities are of length *l* and depth *d* and have a central separation given by the distance *s*.

The approach will be to look at the light rays being emitted by the surface A-B and determining if they can pass through the aperture presented by the left-most annular cavity. If the rays make it through, they will be “collected” and travel the rest of the distance through the fiber to the detector. As the separation *s* is varied, the sector spanned by the angles $\theta_{min}$ and $\theta_{max}$ will change. The power collected, p(s), is assumed to be proportional to the integral across the surface A-B, of the sectors spanned.

(2) $$p\left(s\right)=\int_{0}^{d}\Delta \theta \left(s,x\right)dx=\int_{0}^{d}\left(\theta_{max}\left(s,x\right)-\theta_{min}\left(s,x\right)\right)dx$$

The function Δθ(s,x) is dependent on both the separation of the cavities, as well as the position, *x*, on the surface A-B. The function will have to consider that the maximum value $\theta_{max}$ can take is $\theta_{c}$.

In the analysis, we neglect any reflections that occur on the inner surface of the cavities, as well as the emission from this surface. We also neglect reflections occurring between the two cavities and focus our attention on the “direct” rays passing through the aperture. Our goal is to show an approximative behavior that can clarify the results from the third experiment. In the full numerical model, presented in Section 5, skew rays, reflections at the core-cladding, and the photoluminescence from the inner surface of the cavities are all included.

### 4.1. Dark, Bright, and Blocked Regions

Some of the light entering from the left will pass through the aperture presented by the first cavity and reach the second cavity. Rays hitting the vertical surface of the second cavity will cause photoluminescence to be emitted from that surface, traveling back toward the left. Rays hitting the inner surface of the second cavity will be disregarded in this analysis.

Three regions can exist on the vertical surface. Nearest the cladding, a dark region can exist, where no rays can be drawn through the aperture to a point in the region, without violating the condition that the polar angle be less than $\theta_{c}$ (see Figure 9). The position $x_{dark}$, as measured from Point B on the surface and to the cladding, is the point where this region begins. The region then extends from $x_{d}$ and to Point A. Between $x_{d}$ and $x_{b}$ is the bright region, since rays with polar angles all the way up to $\theta_{c}$ can reach through the aperture. There is still a constriction on the minimum angle of the rays, as can be seen in Figure 9. The region from Point B to $x_{b}$, closest to the core, is termed the partially blocked region, and here, the maximum angle is also constrained by the first cavity.

There are two interesting separations to consider: $s_{dark}$ is the separation at which the dark region disappears and rays can reach the cladding, while $s_{block}$ is the separation at which the partially blocked regions emerges. These two separations are given by the following trigonometric equations:

(3) $$s_{dark}=\frac{d}{\tan(\theta_{c})}+l$$

(4) $$s_{block}=\frac{a}{\tan(\theta_{c})}$$

with a=T−2d being the aperture size presented by the cavity. Likewise, we can calculate the point, $x_{dark}$, on the vertical surface where the boundary between the dark region and the bright region exists, as well as the boundary, $x_{bright}$, between the bright and partially blocked region.

(5) $$x_{dark}=\left\{\begin{array}{cc} \left(s-l\right)\tan\left(\theta_{c}\right) & \mathrm{if}\;s<s_{dark}\\ d & \mathrm{otherwise} \end{array}\right.$$

(6) $$x_{bright}=\left\{\begin{array}{cc} s\tan(\theta_{c})-a & \mathrm{if}\;s\geq s_{block}\\ 0 & \mathrm{otherwise} \end{array}\right.$$

### 4.2. Values Calculated for the Experimental Design

For the two-cavity fibers tested experimentally, the above parameters were calculated and can be seen in Table 1. Using the calculated values, we can determine what kind of behavior we have according to the above. We see that $s_{dark}<s_{block}$, which means that the dark region disappears before the partially blocked region appears.

### 4.3. Radiant Power Integrals

We will now describe the power in terms of the integrals in each of the three sections $s<s_{dark}$, $s_{dark}<s<s_{block}$, and $s>s_{block}$. The integral in Equation (2) can be described in terms of two separate integrals, $p_{b}\left(s\right)$, for the power collected from the bright region, and $p_{p}\left(s\right)$, for the partially blocked region.

(7) $$p\left(s\right)=p_{p}\left(s\right)+p_{b}\left(s\right)=\int_{0}^{x_{b}}\Delta \theta_{p}\left(s,x\right)dx+\int_{x_{b}}^{x_{d}}\Delta \theta_{b}\left(s,x\right)dx$$

As the separation *s* changes, the limits of the integrals will also change. Δθ are given by:

(8) $$\begin{array}{cc} \Delta \theta_{p}\left(s,x\right) & =\arctan\left(\frac{a+x}{s}\right)-\arctan\left(\frac{x}{s-l}\right)=\int_{0}^{x_{b}}\left(\frac{a+x}{s}-\frac{x}{s-l}\right)dx=\int_{0}^{x_{b}}\frac{a}{s}dx \end{array}$$

(9) $$\begin{array}{cc} \Delta \theta_{b}\left(s,x\right) & =\theta_{c}-\arctan\left(\frac{x}{s-l}\right)=\int_{x_{b}}^{x_{d}}\left(\theta_{c}-\frac{x}{s-l}\right)dx \end{array}$$

Since the maximum angle that exists in the problem is 20°, the maximum value of the argument to the arc-tangents will be 0.364. We therefore express them in their Taylor series and keep only the first-order term. In that case, the error at the largest argument will be 4.2%. We likewise make the assumption that s≫l, which is valid in our case where *s* is close to 2 mm and *l* is approximately 130 μm.

#### 4.3.1. When $s<s_{dark}$

Initially, the power collected is described by only the integral for the bright region. Furthermore, $x_{b}=0$, and $x_{d}=\left(s-l\right)\tan\left(\theta_{c}\right)$.

(10) $$p\left(s\right)=\int_{0}^{x_{d}}\left(\theta_{c}-\frac{x}{s-l}\right)dx=\left(s-l\right)\left(\theta_{c}\tan\left(\theta_{c}\right)-\frac{\tan^{2}\left(\theta_{c}\right)}{2}\right)$$

We see that $\theta_{c}$ determines the slope of the function. By inserting the value of $\theta_{c}$ into the equation, the slope is found to be 0.0608 and thus positive.

#### 4.3.2. When $s_{dark}\leq s<s_{block}$

When the separation increases above $s_{dark}=$ 0.52 mm, the integral will change. The upper limit is now $x_{d}=d$, and the integral becomes:

(11) $$p\left(s\right)=\int_{0}^{d}\left(\theta_{c}-\frac{x}{s-l}\right)dx=\theta_{c}d-\frac{d^{2}}{2(s-l)}$$

The interesting part now is that the function will go as 1/s. The larger the separation, the smaller the second negative term in the sum will become and the larger the total collected power.

#### 4.3.3. When $s>s_{block}$

When the cavities are separated even further, the lower part of the surface A-B will begin to be partially blocked by the cavity in front. The partially blocked region emerges, and the integral will change. When s>1.98mm, the total integral becomes:

(12) $$p\left(s\right)=\int_{0}^{x_{b}}\frac{a}{s}dx+\int_{x_{b}}^{d}\left(\theta_{c}-\frac{x}{s-l}\right)dx=\frac{ax_{b}}{s}+\int_{x_{b}}^{d}\left(\theta_{c}-\frac{x}{s}\right)dx=\frac{ax_{b}}{s}+{\left[\theta_{c}x-\frac{x^{2}}{2s}\right]}_{x_{b}}^{d}$$

Because s≫l, the fractional term in the second integral can be approximated by simply x/s, which makes the computation easier. If there is still a bright region ($x_{b}\neq d$), then $x_{b}=s\tan\left(\theta_{c}\right)-a$. This leads to the equation for p(s) given by:

(13) $$p\left(s\right)=-s\left(\theta_{c}\tan\left(\theta_{c}\right)-\frac{\tan^{2}\left(\theta_{c}\right)}{2}\right)-\frac{a^{2}+d^{2}}{2s}$$

By differentiating, we can find the slope of this function and see that it is:

(14) $$\frac{dp}{ds}=-\left(\theta_{c}\tan\left(\theta_{c}\right)-\frac{\tan^{2}\left(\theta_{c}\right)}{2}\right)+\frac{a^{2}+d^{2}}{2s^{2}}=-0.0608+\frac{a^{2}+d^{2}}{2s^{2}}$$

By setting up an inequality, we can find the separation at which the slope becomes negative.

(15) $$\begin{array}{cc} \; & -0.0608+\frac{a^{2}+d^{2}}{2s^{2}}<0 \end{array}$$

(16) $$\begin{array}{cc} \; & s>\sqrt{\frac{a^{2}+d^{2}}{2\;\cdot \;0.0608}}=2052.96=2.05mm \end{array}$$

We therefore find that the total power collected reaches a maximum at 2.05 mm.

### 4.4. Summary

Table 2 summarizes the findings of the previous analysis. It fits well with the experimental data, which show a maximum at around a 2 mm separation. The first range of separation is where the dark region is decreasing and the bright region is increasing. This results in a total increase in the collected power. The next range is entered as the dark region disappears. The full surface of A-B is now illuminated and is a “bright” region, and the overall power increases. At 1.98 mm, a region of partial blocking emerges. The overall power still increases. At 2.05 mm, the partially blocked region has grown big enough to start to reduce the overall collected power, and we see a decrease happening.

## 5. Simulation Model for Annular Cavities

**Figure 10:**
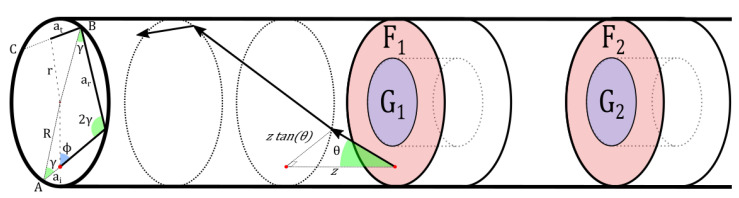
The axial projection plane of the fiber. A ray is emitted from a position in the fiber (red dot) and as an initial direction in the plane given by its azimuthal angle ϕ. The polar angle governs the relationship between the axial-propagation distance, *z*, and the inter-plane distance, a(z), and is given by a(z)=ztan(θ).

To investigate the annular cavity design in more detail, we developed a numerical simulation model. The model is based on the idea that the total collected radiant power of fluorescence emission (units of W) can be described as the sum of the radiant powers from each of four surfaces—F_1_, G_1_, F_2_, and G_2_ (see Figure 10). The F-surfaces are the frontally facing surfaces of the annular cavities, while the G-surfaces are the glazing surfaces of the cavities.

(17) $$P_{col}=P_{F_{1}}+P_{G_{1}}+P_{F_{2}}+P_{G_{2}}$$

Each of the powers can be described as an integral of the collected emittance, *M* (units of $W/m^{2}$), over each surface. By collected, we mean it is the portion of the total emittance, which can make it to the detector. For F-surfaces, the integral becomes:

(18) $$\begin{array}{cc} P_{F_{i}} & =\int_{S}M_{F_{i}}\left(x,y\right)dS=\int_{0}^{2\pi }\int_{\left(R-d\right)}^{R}M_{F_{i}}\left(r,\alpha \right)rdrd\alpha =\int_{0}^{2\pi }\int_{\left(R-d\right)}^{R}rM_{F_{i}}\left(r\right)drd\alpha =2\pi \int_{R-d}^{R}rM_{F_{i}}\left(r\right)dr \end{array}$$

The first step is achieved by converting from Cartesian to cylindrical coordinates for the integration. We assume there is rotational symmetry to the emittance, $M_{F_{i}}$, in such a way that it is independent of the angle, α. This way, we can write the integral as in Equation (18). Similarly, for G-surfaces, we can write an integral. The radiant power for a G-surface also depends on the radius, but the integral is over the length of the G-surface instead.

(19) $$P_{G_{i}}=2\pi r\int_{0}^{L}M_{G_{i}}\left(l\right)dl$$

The collected emittance is itself an integral over the solid angle of the “emission” radiance, $L_{em}$ (units of $W/m^{2}/\mathrm{sr}$), within angles for rays that would be guided by the fiber. This approach considers emitted skew rays. Snyder and Mitchell [44] showed in 1974 that skew rays with a polar angle less than the complementary critical angle $\theta_{c}$ will be totally internally reflected. Skew rays with a polar angle larger than $\theta_{c}$, but an azimuthal angle such that the angle between the ray and the fiber surface normal is within the TIR critical angle, are expected in geometrical optics to be totally internally reflected. However, Snyder and Mitchell showed that these rays are in fact leaky. In our model, we will neglect any rays with polar angles larger than $\theta_{c}$ by assuming that these rays are attenuated completely during the transmission through the fiber optics.

The collected emittance, *M*, can then be found by an integral of the radiance, $L_{em}$, and is expressed in the same way for both F- and G-surfaces. In the integral, *x* is replaced by *r* for F-surfaces and *l* for G-surfaces. In Equation (20), ϕ is the azimuthal angle and θ is the polar angle of the ray. Ω is a differential unit of a solid angle.

(20) $$\begin{array}{c} M\left(x\right)=\int_{\Omega }L_{em}\left(x,\theta ,\phi \right)d\Omega =\int_{0}^{2\pi }\int_{0}^{\theta_{c}}L_{em}\left(x,\theta ,\phi \right)\sin\theta d\theta d\phi \end{array}$$

The difference between the surface F_1_ and F_2_ is that the F_2_ surface is partially blocked by the first cavity, and similarly for G_2_. This means that some combinations of θ and ϕ give zero contribution to the integral, as that direction will be blocked by the cavity in front. This can be modeled as the multiplication of the natural radiance, $l_{em}\left(r,\theta ,\phi \right)$, and a window function W(r,θ,ϕ), where the window function is a function with an output between zero and one. Directions in which the rays from the second cavity hit the “backside” of the first cavity will be completely blocked, and W(r,θ,ϕ) will be zero. Rays that pass directly through the first cavity aperture will travel through the rest of the fiber toward the detector and will correspond to a value of W(r,θ,ϕ)=1. Those rays that hit the inside of the first cavity (that is, they hit the G_1_ surface) may be partially reflected, depending on the model of reflectance used.

For F_1_, there is no window-function, and the emission intensity can be described entirely by the natural radiance. For G_1_, the rays may reflect several times inside the G_1_ cavity before exiting. Depending on the reflection model used for the surface, these rays may be weighted with a factor between zero and one. For the simplest model, in which light rays impinging on the inner surface of the cavities are neglected or assumed lost, the window function is binary and is either zero or one.

The natural radiance originates from within the annular cavity material, and it is dependent on the material, as well as the model used to describe the surface. To be precise, the radiance depends on the projected area of the surface. For a flat surface, the projected area will decrease with the cosine of the viewing angle w.r.t. the surface normal. A Lambertian surface will have equal radiance in all directions, and large viewing angles would have a radiant intensity (units of W/sr) of decreasing magnitude. This is what is behind Lambert’s cosine law. However, for true surfaces, roughness plays a role, and the projected area may have much more complex descriptions. We assume the surfaces to be isotropic emitters, meaning that the projected area remains the same independent of the viewing angle, and the radiant intensity is likewise direction independent.

The power emitted by an F or G surface can be described in terms of a triple integral.

(21) $$\begin{array}{cc} P_{F_{i}} & =2\pi \int_{R-d}^{R}r\int_{0}^{2\pi }\int_{0}^{\theta_{c}}W_{F_{i}}\left(r,\theta ,\phi \right)\;l_{em,F_{i}}\left(r\right)\sin\left(\theta \right)d\theta d\phi dr \end{array}$$

(22) $$\begin{array}{cc} P_{G_{i}} & =2\pi r\int_{0}^{L}\int_{0}^{2\pi }\int_{0}^{\theta_{c}}W_{G_{i}}\left(l,\theta ,\phi \right)\;l_{em,G_{i}}\left(l\right)\sin\left(\theta \right)d\theta d\phi dl \end{array}$$

The natural radiance, of a small surface, is assumed to depend on the irradiance at that point, *E*. This power is similarly described by integrals. In fact, the problem of determining the power of excitation is almost the same as for emission. The integral is over the solid angle from which rays can reach a given differential surface element. The window function plays a role once again, and it is the same as previously described. The relationship between the irradiance *E* and the natural radiance $l_{em}$ is given through a function akin to a Bidirectional Reflectance Distribution Function (BRDF) [45]. It essentially encodes the directionality property of the radiance.

(23) $$l_{em}=f\left(x,y,\phi ,\theta \right)\;\cdot \;E\left(x,y\right)=f\left(x,y,\phi ,\theta \right)\int_{0}^{2\pi }\int_{0}^{\theta_{c}}W\left(x,\theta ,\phi \right)\;l_{ex}\left(x,\theta ,\phi \right)\sin\left(\theta \right)d\theta d\phi$$

The function describes the direction-dependent emission from a surface element and allows for a dependence on position as well, to account for changes in surface characteristics. The irradiance *E* is given by a similar integral as in the previous, considering the radiance, $l_{ex}$, onto the surface. The variable *x* is replaced by *l* in the case of a G surface and *r* in the case of an F surface. The power from F_2_ can thus be described by a quintuple integral:

(24) $$\begin{array}{cc} P_{F_{i}} & =2\pi \int_{R-r}^{R}r\left(\int_{0}^{2\pi }\int_{0}^{\theta_{c}}f\left(r,\theta ,\phi \right)W_{F_{i}}\left(r,\theta ,\phi \right)\sin\theta d\theta d\phi \right)\left(\int_{0}^{2\pi }\int_{0}^{\theta_{c}}W_{F_{i}}\left(r,\theta ,\phi \right)l_{ex}\left(r,\theta ,\phi \right)d\theta d\phi \right)dr \end{array}$$

(25) $$\begin{array}{cc} P_{G_{i}} & =2\pi r\int_{0}^{L}\left(\int_{0}^{2\pi }\int_{0}^{\theta_{c}}f\left(l,\theta ,\phi \right)W_{G_{i}}\left(l,\theta ,\phi \right)\sin\theta d\theta d\phi \right)\left(\int_{0}^{2\pi }\int_{0}^{\theta_{c}}W_{G_{i}}\left(l,\theta ,\phi \right)l_{ex}\left(l,\theta ,\phi \right)d\theta d\phi \right)dl \end{array}$$

Equations (24) and () are the fundamental equations that make up the simulation model. These were implemented in Python/NumPy code.

### 5.1. The Ray Propagation Equations

The window function is central to the problem and can be found by considering the rays being emitted or impinging on a surface element. For the F_1_ surface, there are no obstacles in front of it, and all directions have a value of unity. It therefore suffices to look at the window functions for G_1_, F_2_, and G_2_. To set up the problem, we derive some fundamental equations for the propagation of the rays in the fiber.

A ray being emitted from a surface into the fiber has a radial position *r*, an azimuthal angle ϕ, and a polar angle θ. Another angle exists, γ, which is the angle defining the skewness of the ray. Seen from the cross-sectional projection of the fiber, a ray will be moving in a helical-like path around the fiber circumference. Each time the ray reaches the core-cladding interface, the ray will reflect, and the angle of reflection is γ. Rays with γ=0, corresponding to r=0, are meridional rays and the type of rays most often considered in fiber-optical propagation.

The angle γ for a given ray being emitted is given by a relationship between the position *r* and the azimuthal angle ϕ.

(26) $$\gamma \left(r,\phi \right)=\arcsin\left(\frac{r}{R}\sin\left(\phi \right)\right)$$

During propagation in the fiber and reflections at the core-cladding interface, the polar angle and the propagation distance between reflections, $a_{r}$, remain unchanged [46]. As the ray propagates, its position can be projected to the cross-section of the core. In this cross-sectional plane, its path will look polygonal in nature. At particular propagation distances, the ray may encounter a cavity, and depending on its radial position in this cross-sectional plane (and thus, its distance from the cladding), it may be either lost or pass into the cavity. All the interesting dynamics of the problem therefore depend on the description of the ray propagation in this cross-sectional plane.

As the ray propagates, its total cross-sectional propagation distance, *a*, increases. The relationship between the cross-sectional distance *a* and the axial distance, *z*, is determined by the polar angle.

(27) a(z)=ztan(θ)

As the ray propagates, it will at some point be reflected at the core-cladding boundary. The new angle with respect to the cylindrical surface is again γ, and this angle is conserved throughout the propagation down the fiber. Between any two reflections, the ray behaves as between any other two reflections. The cross-sectional distance between reflections $a_{r}$ can be found by simple trigonometry and is given by:

(28) $$a_{r}=2R\cos\left(\gamma \right)$$

The ray is initially emitted at a radial position of *r* and with an azimuthal angle of ϕ, which together determine the actual skewness angle γ (Equation (26)).

The ray can be seen as having traveled an initial distance $a_{i}\left(r,\phi \right)$ from the “previous” reflection. This initial distance $a_{i}$ is given by:

(29) $$a_{i}\left(r,\phi \right)=\frac{a_{r}}{2}-r\cos\left(\phi \right)=R\cos\left(\gamma \right)-r\cos\left(\phi \right)$$

The radial position of the ray, as it propagates from one reflection point to another, is given by:

(30) $$r\left(z,\theta ,\phi \right)=\sqrt{a_{t}^{2}+R^{2}-2a_{t}R\cos\left(\gamma \right)}$$

In Equation (30), $a_{t}$ is the cross-sectional position of the ray along the line connecting two reflection points. In Figure 10, it corresponds to the line going from Point B toward Point C. It can be found as:

(31) $$a_{t}=a-\lfloor \frac{a}{a_{r}}\rfloor a_{r}=a\;\mathrm{mod}\;a_{r}$$

where the parenthesis denotes the floored division operation. It can also be seen as the modulo, or remainder, operation. The reason for using this approach is that the ray propagates in the same manner between any two reflections. It is therefore only of interest to know the position along the path between two reflections, to know the radial position.

### 5.2. G_1_ Window Function

The G_1_ rays being emitted have the probability of reflecting on the inner surface of the cavity, before exiting and being collected. The number of times a ray has reflected internally can also be computed by the floored division.

(32) $$n\left(l,\phi ,\theta \right)=\lfloor \frac{a}{a_{r,g}}\rfloor =\lfloor \frac{l\tan(\theta )}{a_{r,g}}\rfloor$$

The distance between reflections, $a_{r,g}$, is different inside the cavity region than outside, since the radius of the cylindrical cavity bottom is smaller than the fiber. Its value is computed similarly as above, but with the radius replaced by the radius of the cavity, $R_{cav}=R-d$.

### 5.3. F_2_ Window Function

The rays emitted from the F_2_ surface can be completely blocked by the backside of the first cavity. They can also enter through the aperture presented by the first cavity and then either pass directly through the cavity or be reflected on the G_1_ surface.

Initially, the rays propagate an axial distance given by *s*, which is the separation between the cavities. At the input plane of the first cavity, the radial position of each ray is determined. Those that have a radial position less than $R_{cav}=R-d$ will pass into the cavity, and those with a radial position larger than this will be blocked completely by the cavity.

### 5.4. G_2_ Window Function

To begin with, the G_2_ rays behave similarly to the G_1_ rays. They can reflect a number of times at the inner surface of the cavity. As they exit the cavity region, they will propagate the axial distance *s* to the first cavity and now behave similarly to F_2_, with some rays being completely blocked by the first cavity.

Next, the rays not blocked will pass into the first cavity and reflect a number of times depending on their angle and radial parameters, and finally exit.

### 5.5. Simulation Results

In Figure 5, Figure 6 and Figure 7, we already showed results from model simulations. The model used in those simulations was with the internal cavity reflectance of the G_1_ and G_2_ cavities set to zero (and therefore, all rays hitting a G-surface would be totally lost). Additionally, they were made with a radial power distribution of one, that is no matter the direction and no matter the radial position, the power in the excitation ray would be the same.

Such a model is obviously very simplified, but the important thing to notice is that the general trends can be seen. The collected power rises as we increase both the cavity depth or length, but with the gain diminishing for longer lengths. This hints at the idea of self-blocking from the first cavity being the reason behind the smaller gain with longer lengths seen in the experimental results. The simulation for the separation of two cavities also shows a comparable trend to the experimental findings, but the gain at 2 mm is greatly undershot.

**Figure 11:**
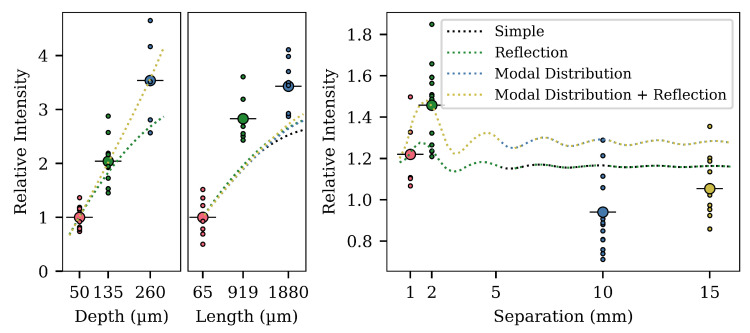
Simulation results with different combinations of effects added to the model. The intensity is relative to the intensity from a single cavity with the same geometrical parameters as either of the two annular cavities in the dual-cavity fibers (l= 0.15 mm and d= 125 μm). The black dotted curve is the same model as used in Figure 5, Figure 6 and Figure 7 and is the simplest model, with an excitation function of unity. The black curves are hidden behind the green curves on the left-most and right-most plots, as adding reflection only has minimal influence on the effect of separation. The same is true for the blue and yellow curves, where the blue is hidden behind the yellow in the left and right plots. The intensity at 2 mm separation can also be quantified relative to a single cavity with double the length. In that case, the relative intensity is 1.2.

In Figure 11, we see the three experiments once again. Each curve is a simulation of the model, with a different combination of effects included. Adding simple reflectance to the G-surfaces changes the results of the simulations for varying lengths and depths of a single cavity; however, it does little to change the results for the double cavity simulation. If we instead account for a distribution in the ray power given by the equation:

(33) $$p\left(\gamma \right)=e^{-{\left(\gamma /w\right)}^{b}}$$

then we see a large change in the simulation of the dual cavities. In the function, *w* is a width parameter and *b* is an exponent signifying how super-Gaussian the function is (b=2 is a standard Gaussian function). In the simulation, the values were chosen as w=0.4 and b=6. It seems likely that the simulation must consider the power distribution in the rays, and more complex models could be generated. However, we do not know the correct distribution, and so, we have simply fit the parameters as best we could to show the effect.

The results from the simulations and the analytical expressions indicate that the trend of having a maximum arises from a consideration of the window functions for the surfaces F_1_, G_1_, F_2_, and G_2_. As the separation is increased, the window functions will change, and depending on the geometry of the cavities, the optimum will change.

The model simulation for the separation had a discrepancy in relation to the experimental values. The optimal value was found to lie lower than experimentally at a 2 mm separation. However, the analytical part of this paper described an optimal separation of 2 mm, when considering only meridional rays. The reason behind the discrepancy between the experiment and the simulation may be in the incorporation of skew rays. The meridional analysis only considered meridional rays, and the contribution to the power from the skew rays was zero. In the simulation, equal weight was given to meridional and skew rays. The correct model may have to account for differences in the power being radiated in skew and meridional rays. Another large factor that will have an impact on the results is the surface model used. When a light ray hits the inner surface of a cavity, a model of the surface and the scattering has to be assumed, in order to explain how the light is reflected and scattered due to both specular and diffuse reflection. One approach to this may come from applying a Bidirectional Scattering Distribution Function (BSDF) [45].

Another factor that has not been included is the material losses in the fiber. Rays with skew trajectories will travel a much longer distance than rays that are more directly oriented along the fiber axis, which favors the meridional analysis results. Material loss through absorption will lead to a smaller contribution to the total radiated power of skew rays, which again may lead to a better model fit.

## 6. Conclusions

We presented results on the design of a photoluminescent dual-annular cavity POF sensor and showed that the photoluminescent response varies with depth, length, and cavity separation. It was found that an optimal separation exists, for which the photoluminescent response is greatest, and we proposed an analytical answer to this using a ray optic meridional analysis. A simulation model considering both skew and meridional rays was also constructed and could to some degree verify the trends we saw in the experimental results for depth, length, and cavity separation. The work has potential for paving the way to the design of multi-point photoluminescent sensors, where optimizing for the maximally received signal from the sensing points will be important.

It is possible to achieve a strong photoluminescent response by creating a long cavity, but it becomes more difficult to fill this cavity with an even layer, as the length is increased. We instead create a short cavity, which will allow the gel to creep into and around the cavity by itself—due to surface tension. Creating such a short cavity will come at the expense of signal strength, and so, we proposed a solution to this trade-off. By adding a second cavity, closely after the first one, it is possible to achieve a photoluminescence signal equal to or greater than a single cavity of double the length. It is possible to choose the distance such that an optimal intensity can be collected. With a separation of 2 mm, we achieved an increase in signal strength of 1.2, compared to the intensity of a single cavity with the same axial length as the two annular cavities combined.

Our experiments, analyses, and numerical investigations all provide evidence for the suitability for annular cavities as a general platform for multipoint sensing of several measurands.

## Figures and Tables

**Table 1:** Two-cavity fiber parameters.

Parameter		Value
*d*	average cavity depth	140 μm
*l*	average cavity length	130 μm
$s_{dark}$	dark region disappears	0.52 mm
$s_{block}$	emergence of partially blocked region	1.98 mm

**Table 2:** Effects at critical separations.

Separation (mm)	Effect Until This Point
0–0.52	Decreasing dark region. Increasing collected power.
0.52–1.98	Dark region disappears. Overall increase in power.
1.98–2.05	Decreasing bright region. Increasing blocked region. Overall increase in power.
2.05 +	Decreasing collected power.
